# Supplementary figures and images for: Community engaged tick surveillance and tickMAP as a public health tool to track the emergence of ticks and tick-borne diseases in New York
Source: PLOS Glob Public Health. 2022 Jun 27;2(6):e0000215. doi: 10.1371/journal.pgph.0000215 (PMC10022224; doi:10.1371/journal.pgph.0000215)

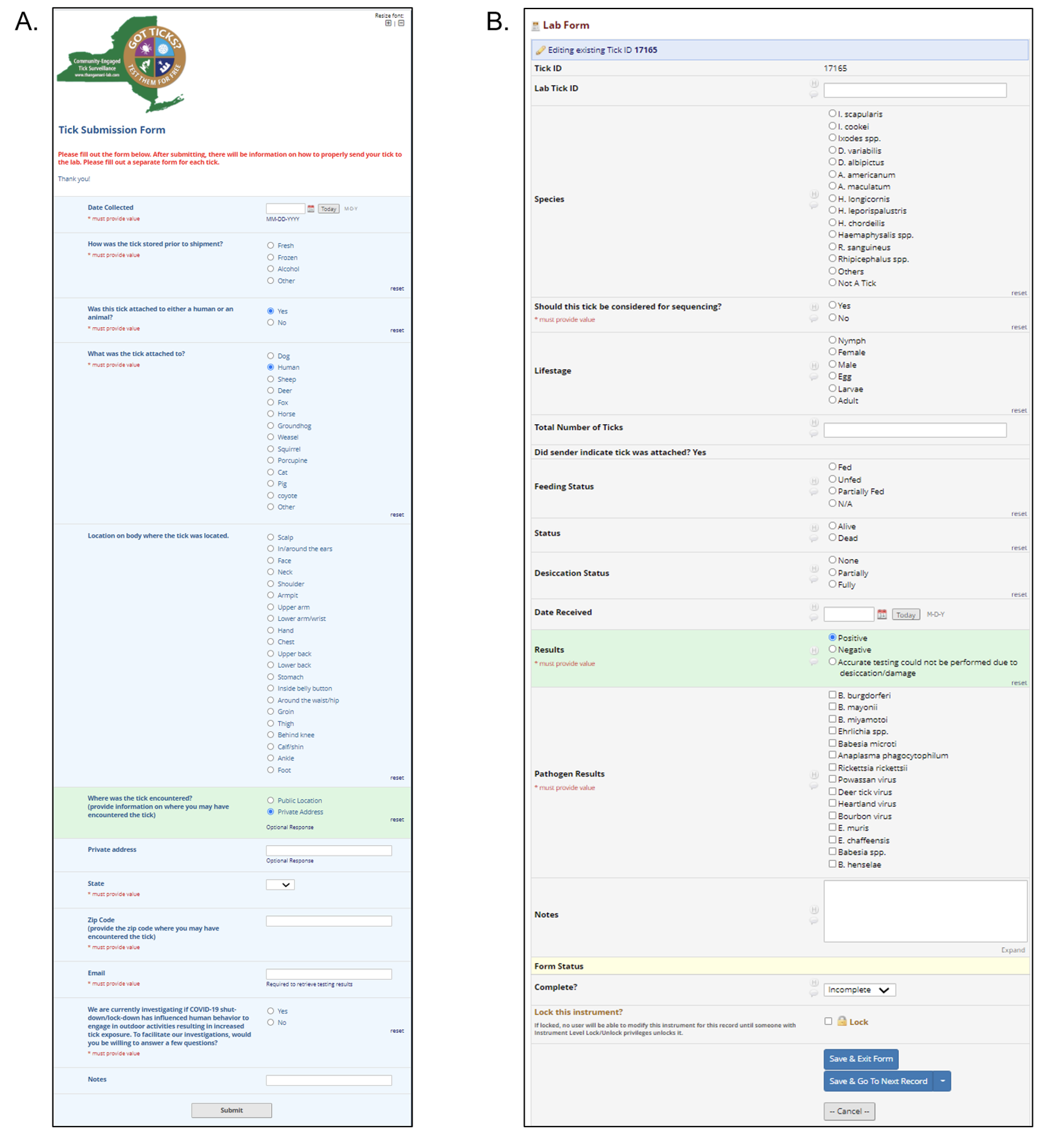

Supplement: S1 Fig — Two-part survey in REDCap. Tick Submission Form (A), completed by tick submitters, and the Lab Form (B), completed by technical staff. (TIFF) [file pgph.0000215.s001.tiff]
