# Supplementary material for: Community engaged tick surveillance and tickMAP as a public health tool to track the emergence of ticks and tick-borne diseases in New York
Source: PLOS Glob Public Health. 2022 Jun 27;2(6):e0000215. doi: 10.1371/journal.pgph.0000215 (PMC10022224; doi:10.1371/journal.pgph.0000215)
Supplement: S1 Table — Efficiencies and r2 values for the primers in multiplex panels A and B, as determined by standard curves using known numbers of target-sequence DNA molecules for each pathogen. (DOCX) [file pgph.0000215.s002.docx]

**S1 Table:** Efficiencies and r^2^ values for the primers in multiplex panels A and B, as determined by standard curves using known numbers of target-sequence DNA molecules for each pathogen.

| **Panel** | **Target** | **Efficiency** | **R^2^** |
| --- | --- | --- | --- |
| A | *B. microti* 18s | 93.82% | 0.987 |
|  | *A. phagocytophlium* msp2 | 98.98% | 0.997 |
|  | *B. miyamotoi* FlaB | 100.12% | 0.991 |
|  | *B. burgdorferi* OspA | 101.37% | 0.978 |
| B | Heartland S-segment | 87.20% | 0.991 |
|  | Bourbon PB1 | 98.43% | 0.994 |
|  | *E. muris eauclariensis* P13 | 94.34% | 0.984 |
|  | *E. chaffeensis* DSB | 102.96% | 0.998 |
|  | DTV NS5 | 113.3% | 0.964 |
